# A DNA-Damage Inducible Gene Promotes the Formation of Antibiotic Persisters in Response to the Quorum Sensing Signaling Peptide in *Streptococcus mutans*

**DOI:** 10.3390/genes13081434

**Published:** 2022-08-12

**Authors:** Delphine Dufour, Haowei Zhao, Siew-Ging Gong, Céline M. Lévesque

**Affiliations:** Faculty of Dentistry, University of Toronto, Toronto, ON M5G 1G6, Canada

**Keywords:** persister gene, DNA damage, quorum sensing, streptococci, toxin–antitoxin, GG-type leader bacteriocin

## Abstract

Bacteria use quorum sensing (QS) to communicate with each other via secreted small autoinducers produced by individuals. QS allows bacteria to display a unified response that benefits the species during adaptation to environment, colonization, and defense against competitors. In oral streptococci, the CSP-ComDE QS is an inducible DNA damage repair system that is pivotal for bacterial survival. In the oral pathogen *Streptococcus mutans*, the QS system positively influences the formation of antibiotic persisters, cells that can survive antibiotic attack by entering a non-proliferative state. We recently identified a novel gene, *pep299*, that is activated in the persister cell fraction induced by QS. In this study, we focused our investigation on the role of *pep299*, a gene encoding a bacteriocin-like peptide, in the formation of antibiotic persisters. Mutant Δ299, unable to produce Pep299, showed a dramatic reduction in the number of stress-induced persisters. Using a co-culture assay, we showed that cells overproducing *pep299* induced the formation of persisters in the mutant, suggesting that Pep299 was actively secreted and detected by neighboring cells. Cells exposed to DNA damage conditions activated the gene expression of *pep299*. Interestingly, our results suggested that the *pep299* gene was also involved in the regulation of a QS-inducible toxin–antitoxin system. Our study suggests that the *pep299* gene is at the core of the triggered persistence phenotype in *S.* *mutans*, allowing cells to transition into a state of reduced metabolic activity and antibiotic tolerance.

## 1. Introduction

Bacteria are able to communicate with one another using small hormone-like molecules, a process called quorum sensing (QS) [1]. For most human pathogens, QS is essential for the establishment of a pathogenic relationship with eukaryotic hosts [2]. Although many QS systems provide cues about bacterial strategies that have evolved for communication, many molecules remain to be discovered and the roles and functions of QS in natural ecosystems yet to be elucidated [3]. The oral pathogen *Streptococcus mutans* communicates using a canonical Gram-positive intracellular QS system, the CSP-ComDE [4]. This system is composed of the secreted peptide, the CSP pheromone, and the ComDE two-component system. The CSP pheromone accumulates in the external environment and upon reaching a specific threshold concentration, directly interacts with the membrane-bound ComD receptor initiating a phosphorylation cascade leading to the activation of the cytoplasmic response regulator ComE for regulation of the CSP regulon [5]. Initially discovered and characterized in *Streptococcus pneumoniae*, the CSP-ComDE QS system allows bacteria to regulate the development of genetic competence for the uptake of eDNA [6]. In *S. mutans*, activated ComE directly activates the expression of several genes encoding bacteriocins and bacteriocin-like peptides [7,8]. Bacteriocins are small antimicrobial peptides used by producing cells to kill competitors or closely related bacterial species for limited resources and niche space [9]. Streptococci also performs bacteriocin-mediated killing to get access to the diverse pool of eDNA present in their ecological niche [10]. There are several examples of bacteriocins exhibiting a bifunctional role with antimicrobial and signaling activities. Some bacteriocins are found to play regulatory functions, including their own production [5,11,12]. In *S. mutans*, for instance, mutacin V acts as a signaling peptide to regulate the expression of a murein hydrolase to promote cell lysis [13]. Two types of bacteriocins have been characterized in *S. mutans*, the post-translationally modified lantibiotics and the unmodified peptides, or nonlantibiotics [14]. In contrast to the lantibiotic bacteriocins, which are produced by only a small minority of *S. mutans* strains, the nonlantibiotics are present in every isolate [15]. Generally, the nonlantibiotic bacteriocins produced by *S. mutans* are regulated by the CSP-ComDE QS system and most of them are synthesized as propeptides containing a double glycine (GG) secretion motif in their leader sequence [7,14,16].

A fundamental property of bacteria is their ability to regulate cell growth under adverse conditions. Stress conditions such as UV radiation or oxidative radicals induce the SOS response, a transcriptional regulatory mechanism to address DNA damage [17]. Interestingly, global transcriptome analysis of CSP-regulated genes in *S. mutans* and *S. pneumoniae* showed that DNA damage could activate the CSP-ComDE pathway to promote recombinational mechanisms for the repair of damaged DNA [18,19]. Previous studies showed that streptococci lack the classical SOS response system [20]. In this context, the CSP-ComDE system is more likely part of a larger stress–response regulon with CSP acting as a stress-inducible alarmone enabling cells to protect themselves from DNA damage [18,21,22].

Work performed by our group has shown that the CSP pheromone positively influences the formation of antibiotic persisters [23]. Persisters are phenotypic variants within a clonal antibiotic-susceptible population (“normal” cells) that can survive through growth arrest exposure to lethal doses of antibiotics [24,25]. Antibiotic persisters are not resistant mutants as they cannot replicate in the presence of the antibiotic. By entering a non-proliferative state, persisters are shutting down the activity of essential cellular processes targeted by antibiotics, allowing them to survive. Persisters are thus an extremely antibiotic-recalcitrant subpopulation of bacterial cells. Experimentally, this phenotype is characterized by a biphasic killing curve, the hallmark of antibiotic persisters, showing the rapid killing of nonpersisters until reaching a survival plateau of tolerant persisters [26]. Persisters constitute a small fraction of stationary phase cultures but become a significant fraction (up to 1%) in biofilms [27]. Persisters can regrow when favorable conditions are restored, causing relapse or sustained chronic bacterial infections [28]. Although being non-proliferating, persisters are primed for metabolic uptake and central metabolism [29]. Persisters represent thus a reservoir of viable bacteria that could acquire antibiotic resistance by horizontal gene transfer [30].

We previously demonstrated that DNA damage increased the level of antibiotic persisters in *S. mutans* [23,31]. However, this stress-inducible persistence phenotype was abolished in all QS-deficient mutants unable to produce (Δ*comC*), detect (Δ*comD*) and/or respond (Δ*comE*) to the CSP pheromone [31]. In *S. mutans*, the CSP-ComDE QS system plays a crucial role in the regulation of genes encoding bacteriocins and related peptides. Therefore, we hypothesized that the enhanced antibiotic tolerance can in part traced back to up-regulated bacteriocin genes specifically activated in persister bacteria. To explore this possibility, we performed transcriptomic analysis and identified a bacteriocin gene, named *pep299*, whose expression increased with persister enrichment and thus was classified as specific persister gene. In the present study, we sought to establish a role for *pep299*, which is annotated as a putative GG-leader bacteriocin gene.

## 2. Materials and Methods

### 2.1. Bacterial Culturing and Genetic Manipulations

The storage of bacterial stocks was done in nutrient-rich medium supplemented with 15% (*v/v*) glycerol and stored at −80 °C. Streptococcal strains were routinely cultivated in Todd-Hewitt broth (BD Difco) supplemented with 0.3% (*w/v*) yeast extract (THYE) at 37 °C with 5% CO_2_. *E. coli* DH10B cells were grown in Luria Bertani medium with aeration at 37 °C. When necessary, medium was supplemented with chloramphenicol (20 µg/mL) for *E. coli* and chloramphenicol (10 µg/mL), erythromycin (10 µg/mL) or spectinomycin (1 mg/mL) for *S. mutans*. A previously described nonpolar allelic replacement technique was used to generate deletion mutants in *S. mutans* UA159 (hereafter called the wild-type (WT) strain) [32] using an antibiotic (erythromycin or spectinomycin) resistance cassette. The *E. coli*–*Streptococcus* shuttle plasmid pIB166 [33] was used for the construction of vectors for ectopic expression. Briefly, the full-length coding region of *pep299* or *tox40* was PCR amplified using WT genomic DNA as the template. The PCR products were double digested (SacII/EcoRI) and cloned into the pIB166 vector. Plasmids were introduced into *E. coli* by transformation using electroporation. DNA (circular, linear) was transferred into *S. mutans* by natural transformation [34]. Table 1 lists the mutant strains constructed in this study using the WT strain. The oligonucleotide sequences used in this study are shown in Table S1.

### 2.2. Preparation of Inoculum for Persister Assays

A standardized procedure for the preparation of inoculum was used as described by Kaldalu et al. [35]. Briefly, a few freshly grown colonies of *S. mutans* were picked and added to THYE broth (3 mL) for overnight culture in a test tube. The culture was diluted (1:20) into fresh THYE broth and incubated statically at 37 °C until an optical density of 0.5 at 600 nm was reached. The culture was then supplemented with 15% (*v/v*) glycerol, dispensed in 350 µL aliquots in screw cap microtubes and stored frozen at –80 °C. For the persister assay, an overnight culture was prepared by melting 100 µL of the aliquot into 3 mL of fresh THYE. Frozen aliquots were not refrozen and reused.

### 2.3. Persister Assays

For the classical persister assay, overnight bacterial cultures (20 h) were diluted (1:20) into 5 mL of fresh THYE broth supplemented with 10× MIC of ofloxacin antibiotic followed by incubation for 24 h at 37 °C to kill all nonpersisters. Samples withdrawn at the introduction of the antibiotic (time 0) and at the indicated times were washed once with phosphate-buffered saline (PBS), serially diluted, and plated onto THYE agar plates. Colonies were counted after 48 h of incubation at 37 °C and the percentage of persisters was determined by CFU counts.

For the CSP persister assay, overnight bacterial cultures (20 h) were diluted (1:100) into 5 mL of fresh THYE broth with or without 50 ng/mL of CSP pheromone and incubated for 2 h at 37 °C [23]. Cells were then treated with 10× MIC of ofloxacin for 24 h at 37 °C. The level of persisters in the presence (‘persisters-CSP’) or absence (‘persisters-NC’) of CSP was determined by comparing the number of surviving cells spot-plated onto THYE agar plates.

For the biofilm persister assay, static biofilms were first developed in polystyrene microtiter plates as described previously [23]. The planktonic cells were carefully removed, and the biofilm cells left intact in the wells were rinsed once with PBS to remove loosely bound cells. The biofilms were detached by physical scraping and collected by centrifugation. Biofilm cells were resuspended into fresh THYE broth and treated with 50× MIC of ofloxacin for 24 h at 37 °C to kill all nonpersisters. The percentage of persisters was determined by CFU counts as described above.

For the co-cultivation persister assay, individual overnight bacterial cultures were adjusted to the same optical density and diluted into fresh THYE broth at 1:100 (monoculture) or 1:200 (coculture) and incubated for 2 h at 37 °C in the presence of CSP (‘persisters-CSP’) or absence of CSP (‘persisters-NC’). The samples were then treated with 10× MIC of ofloxacin for 24 h at 37 °C. The percentage of persisters was determined by CFU counts as described above.

For the persister assay using the synthetic Pep299 peptide (S-Pep299), overnight bacterial cultures (20 h) were diluted (1:100) into 200 µL of fresh THYE broth per well with or without 5 µg/mL of S-Pep299 (Shanghai Royobiotech, Ltd., Shanghai, China) in 96-well polystyrene microtiter plates and incubated for 2 h at 37 °C. Cells were then treated with 10× MIC of ofloxacin for 24 h at 37 °C. The percentage of persisters was determined by CFU counts as described above.

### 2.4. Antimicrobial Activity Analysis

A spot-on-lawn assay was performed by drop testing S-Pep299 (0.2–30 nM) on top of a Columbia agar (BD Difco) overlay containing the sensitive target strain (*Micrococcus luteus*, *Lactococcus lactis*) at approximately 10^7^ CFU/mL. Plates were incubated overnight at 37 °C, after which they were examined for evidence of growth inhibition. Using a deferred antagonism assay, the Δ299 mutant was tested for its patterns of antimicrobial activity against a panel of nine standard indicators as previously described [36,37].

### 2.5. Isolation of Persisters-CSP for Gene Expression Analysis

Overnight bacterial cultures (20 h) were diluted (1:100) into fresh THYE broth and incubated for 2 h at 37 °C in the presence of CSP (‘persisters-CSP’). Bacterial cells were then exposed to 10× MIC of ofloxacin for 24 h at 37 °C to kill all nonpersisters. Controls without ofloxacin included CSP-induced cultures (normal-CSP) and no CSP cultures (normal-NC). Cells were harvested by centrifugation, washed once with PBS before being processed for total RNA extraction.

### 2.6. DNA Damage for Gene Expression Analysis

Overnight bacterial cultures were diluted (1:100) into fresh THYE medium until the mid-log phase was reached. Bacterial cells were then exposed for 2 h at 37 °C (except for heat, 50 °C for 30 min) to ofloxacin, hydrogen peroxide, or mitomycin C at lethal concentrations [31]. Bacterial cells harvested by centrifugation were washed once with PBS before being processed for total RNA extraction.

### 2.7. RNA Isolation and Gene Expression Analysis

Bacterial cells were collected by centrifugation and resuspended into ice-cold RNAwix solution (Ambion) containing 0.1 mm ice-cold Zirconia beads. Following cell disruption, total RNA was extracted using the RiboPure-Bacteria purification kit (Ambion) according to the manufacturer’s recommendations. Total RNA was DNAse-treated with RQ1 DNase (Promega) and converted to cDNA using a High-Capacity cDNA Reverse Transcription Kit (Applied Biosystems, Waltham, MA, USA). Quantitative Real-Time PCR (qRT-PCR) analysis was performed using Forget-Me-Not EvaGreen qPCR Master Mix (Biotium) and the CFX96 real-time PCR detection system (Bio-Rad). Data analysis was performed using relative quantification normalized against unit mass.

### 2.8. Statistical Analysis

All assays were carried out in triplicate and data analyzed using the unpaired *t*-test with statistical difference defined as *p* < 0.05.

## 3. Results

### 3.1. A Bacteriocin Gene Is Specifically Activated in the Antibiotic Persister Population

**Figure 1:**
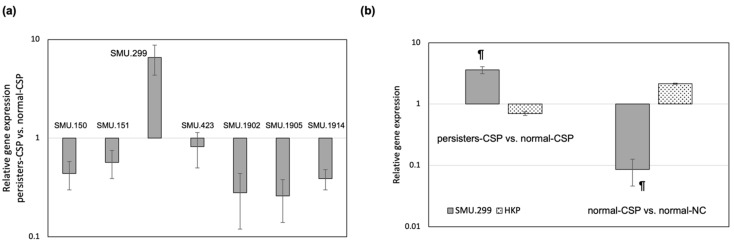
Gene expression analysis by qRT-PCR. (**a**) Relative expression level of putative GG-type leader bacteriocin genes in persisters-CSP. SMU number corresponds to NCBI designation for that particular ORF in the reference sequence (AE014133.2). Bacterial cells were incubated with the CSP pheromone for 2 h and treated with ofloxacin for 24 h to kill all nonpersisters. Control cultures were normal-CSP cells. (**b**) Relative expression level of gene SMU.299. The gene encoding the gyrase subunit B was used as housekeeping (HKP) control gene. The data are the average of three independent experiments ± standard error. ^¶^ denotes statistical significance between SMU.299 and HKP under the same conditions.

Previous studies on the transcriptional response of *S. mutans* to the QS CSP pheromone revealed that the GG-type leader bacteriocin genes were upregulated in the early phase of the CSP response [38]. To further investigate this phenomenon, we analyzed the expression of the bacteriocin genes in the CSP-enriched antibiotic persister population. We targeted the genes encoding mutacin IV (SMU.150-151), mutacin V (SMU.1914), mutacin VI (SMU.423), and three putative bacteriocin genes (SMU.299; SMU.1902; SMU.1905) identified by BAGEL4 [39] search, a web-based bacteriocin server freely available at http://bagel4.molgenrug.nl [39]. We conducted qRT-PCR to measure the expression of the selected genes in the persisters-CSP population vs. the normal-CSP population. As shown in Figure 1a, all the targeted genes but one were found to be repressed in the persisters-CSP population. Of particular interest was the gene SMU.299, which was found highly expressed (approximately seven-fold) in the persisters-CSP. To determine if gene SMU.299 was specifically activated in the persister population, we investigated the expression in normal cells induced with CSP vs. normal cells without CSP. The gene encoding the gyrase subunit B was used as a housekeeping (HKP) control gene for expression in normal replicating cells. As illustrated in Figure 1b, SMU.299 was found activated in the persisters-CSP but not in the normal-CSP population. Opposite results were obtained for the control HKP gene. As expected, HKP was activated in normal-CSP but was found suppressed in the persisters-CSP cells indicating that persistence in *S. mutans* is the opposite of an actively multiplying state.

### 3.2. SMU.299 Is a DNA-Damage Inducible Gene

Previous work conducted by some members of our group showed that DNA damage conditions could positively influence the production of antibiotic persisters in a QS-dependent manner [23,31]. Since the SMU.299 gene was specifically activated in the CSP-enriched persister population, we monitored its expression following DNA damage conditions known to induce persister formation. The levels of SMU.299 transcripts were found to be significantly upregulated by all DNA damage conditions tested: ofloxacin (46.25 ± 8.02), heat shock (12.95 ± 0.32), mitomycin C (3.15 ± 0.10), and hydrogen peroxide (1.73 ± 0.18). These results suggest that the gene SMU.299 is a DNA damage-inducible gene.

### 3.3. Pep299 Does Not Possess Bacteriocin Activity

Bioinformatics analysis showed that SMU.299 (denoted as *pep299* hereafter) encodes a conserved peptide of 72 aa residues in length with sequence homology to the class II bacteriocin garvicin Q (GenBank: AAN58063.1), a nonlantibiotic (class II bacteriocin) with a relatively broad antimicrobial spectrum [40]. Bioinformatics analysis showed that Pep299 contains a leader sequence for excision by an N-terminal peptidase C39 domain at a double Gly motif with conserved hydrophobic residues at positions −4, −7, and −12 distal to the cleavage site (theoretical mass of 5171.63 Da after cleavage). The predicted mature form of the peptide was custom synthesized (Shanghai Royobiotech, Ltd.), and the mass was confirmed by MALDI-TOF. The synthetic peptide, S-Pep299, was examined in growth inhibition assays using the indicator strains *M. luteus* and *L. lactis*, two bacterial species commonly used in bacteriocin assays [41]. S-Pep299 did not exhibit any antimicrobial activity at concentrations ranging from 0.2 to 30 nM using the spot-on-lawn assay [36]. The gene encoding Pep299 was also deleted in the *S. mutans* UA159 WT strain and the antimicrobial activity of its Δ299 mutant was tested using a deferred antagonism assay against a panel of nine standard indicator strains [36,37]. Inactivation of *pep299* did not appear to affect the inhibitory activity of *S. mutans* against all tested strains (data not shown). Altogether, these results suggested that *pep299* may encode a signaling peptide for QS and/or crosstalk mechanisms instead of a toxic peptide for antagonistic activity.

### 3.4. The CSP-Inducible Persistence Phenotype Is Abolished in a Mutant Deficient in Pep299

**Figure 2:**
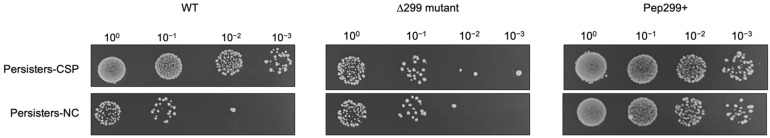
Formation of *S. mutans* antibiotic persisters. Cultures of wild-type (WT), Δ299 mutant, and Δ299-overexpressing mutant (Pep299+) were incubated with CSP (‘persisters-CSP’) or without CSP (‘persisters-NC’) before being treated with ofloxacin antibiotic to kill all nonpersisters (see Methods for details). Cells were washed with PBS, serially diluted (10^0^; 10^−1^; 10^−2^; 10^−3^), and spot-plated onto THYE agar. Pictures shown are representative of three independent experiments.

The Δ299 mutant was next assayed for formation of antibiotic persisters in the presence or absence of the QS CSP pheromone. For the formation of persisters-CSP, *S. mutans* cells were exposed to the pheromone before being treated with ofloxacin antibiotic to kill all nonpersisters. As expected, the WT strain produced a significant >10-fold increase in the number of persisters (‘persisters-CSP’) following induction with the pheromone vs. no-CSP control (‘persisters-NC’) (Figure 2). Interestingly, the CSP-inducible persistence phenotype was abolished in the Δ299 mutant subjected to CSP exposure. In contrast, deletion of *pep299* gene did not affect the persister formation under the no-CSP condition, reinforcing the hypothesis than more than one single mechanism is responsible for persister formation [42]. The fact that Δ299 mutant cells produced significantly less (>10-fold decrease) persisters when compared to the WT strain under CSP conditions suggested a role for *pep299* in stress-activated persister formation.

We next tested the direct impact of *pep299* in the development of antibiotic persisters using mutant cells overexpressing the *pep299* gene. The full-length coding region of Pep299 was cloned in the shuttle plasmid pIB166 for constitutive expression and the construct (Pep299+) was tested using our CSP persister assay. Our results showed that the Pep299+ strain produced substantial numbers of persisters. In fact, the number of antibiotic persisters did not change in Pep299+ even if the cells were preincubated with the CSP pheromone before the antibiotic treatment. These results suggested that *pep299* gene may be responsible for the high persistence phenotype observed during CSP induction.

### 3.5. Pep299 Enhances the Formation of Persisters in Biofilms

**Figure 3:**
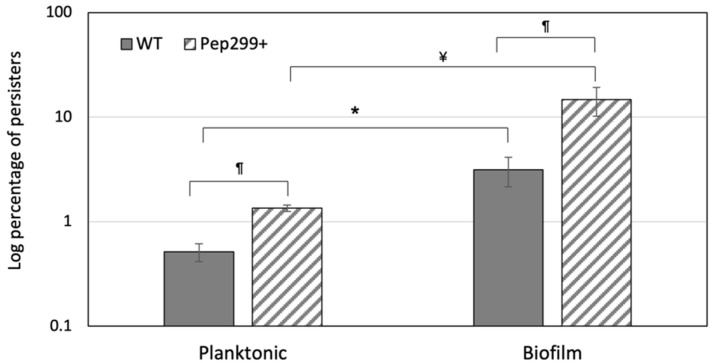
Formation of persisters by biofilm and planktonic cells. Static biofilms were developed using a microtiter plate. The biofilm and planktonic cells were treated for 24 h with ofloxacin to kill all nonpersisters. Aliquots of cells were removed at the introduction of the antibiotic (T = 0) and after the antibiotic treatment (T = 24). Cells were washed with PBS, serially diluted, and spot-plated onto THYE agar for CFU determination. The data (normalized to T = 0) are the average of three independent experiments ± standard error. ^¶^ denotes statistically significant difference between Pep299+ and WT under the same conditions. * denotes statistically significant difference between WT biofilm and WT planktonic. ^¥^ denotes statistically significant difference between Pep299+ biofilm vs. Pep299+ planktonic.

We previously showed that *S. mutans* produces higher numbers of persisters as the biofilm matures, probably due to the accumulation of CSP within the biofilm [23]. Therefore, we next sought to determine whether the overexpression of *pep299* could produce even higher numbers of antibiotic persisters in a biofilm. Using crystal violet staining, we first confirmed that deletion of *pep299* gene or its expression did not affect the biofilm biomass under the conditions tested (data not shown). We next performed a biofilm persister assay using static biofilms developed in a microtiter plate. As expected, the level of antibiotic persisters was statistically significantly greater (greater than five-fold) in the biofilm phase when compared to the free-floating planktonic phase for the WT strain (Figure 3). The Pep299+-overexpressing mutant produced even higher levels of antibiotic persisters (≥10-fold) in the biofilm vs. planktonic cultures. As shown in Figure 3, the levels of persisters in Pep299+ biofilms represent ~15% of the bacterial population. Altogether, these results suggested that *pep299* functions as a persister-gene-promoting bacterial persistence factor of *S. mutans* during planktonic and biofilm growth.

### 3.6. Secreted Pep299 Promotes the Formation of Antibiotic Persisters in Neighboring Cells

**Figure 4:**
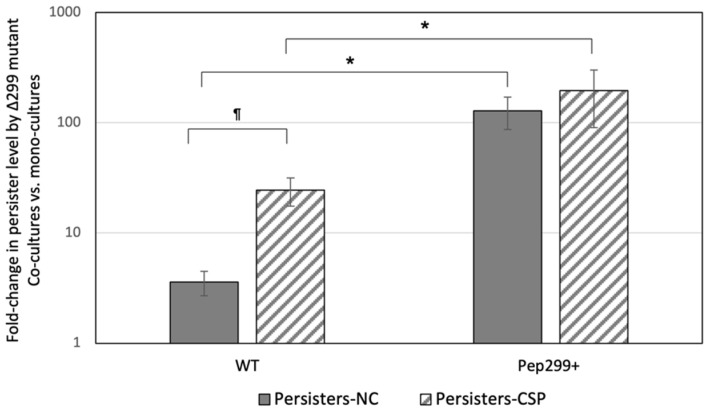
Effect of *pep299* on persister formation by neighboring Δ299 mutant cells. Δ299 mutant was co-cultivated for 2 h with a *S. mutans* wild-type strain harboring an empty plasmid (WT) or a Δ299-overexpressing mutant (Pep299+) in the presence (‘persisters-CSP’) or absence (‘persisters-NC’) of CSP. The co-cultures were then treated with ofloxacin for 24 h to kill all nonpersisters. Monocultures of Δ299 mutant were used as a control. Aliquots of cells were removed at the introduction of the antibiotic (T = 0) and after the antibiotic treatment (T = 24). Samples were washed with PBS, serially diluted, and spot-plated onto THYE agar for CFU determination. The data are the average of three independent experiments ± standard error. ^¶^ denotes statistically significant difference between WT ‘persisters-CSP’ vs. WT ‘persisters-NC’. * denotes statistically significant difference between Pep299+ vs. WT under the same conditions.

Since the *pep299* gene promotes the formation of antibiotic persisters in planktonic cultures and high-cell density biofilms, we hypothesized that Pep299 peptide released by producing cells could also act on nearby cells unable to produce the peptide themselves and increase the formation of antibiotic persisters. To explore this possibility, we tested the formation of antibiotic persisters by the Δ299 mutant cultivated in the presence of the WT strain or Pep299+-overexpressing mutant. The experiments were performed in the presence or absence of the CSP pheromone. As shown in Figure 4, the formation of antibiotic persisters by the Δ299 mutant was increased (approximately four-fold) when co-cultivated with the WT strain. The presence of the CSP pheromone produced even higher numbers with a fold increase of approximately 25-fold. The strongest induction (~150-fold) was obtained when the Δ299 mutant was cultivated in the presence of the Pep299+ strain. Since *pep299* gene is most likely responsible for the high persistence phenotype observed during the CSP induction (Figure 2), the number of persisters did not change when Δ299/Pep299+ co-cultures were supplemented with CSP, as overexpression of the *pep299* gene can bypass the need for the induction of the gene by the pheromone. Cultures of Δ299 mutant were also treated with the S-Pep299 peptide. As expected, the formation of antibiotic persisters was increased (≥10-fold) in Pep299-treated cells vs. untreated cells suggesting a role for Pep299 peptide in cell signaling for the induction of the antibiotic persistence phenotype.

We thus sought to determine if Pep299 was a diffusible peptide or remained mainly attached to the cells after secretion. Preliminary work using a two-chamber system was performed. In this system, Pep299+ and Δ299 mutant cells were separated by a semipermeable membrane. Our results confirmed the ability of Pep299 to diffuse and stimulate the production of antibiotic persisters in Δ299 mutant cells, although at lower levels. Although the findings are preliminary, these results suggest that Pep299 is partly released in the surroundings, while some remains attached to the bacteria.

### 3.7. Pep299 Induces Persister Formation via the Activation of a Specific Toxin-Antitoxin System

**Figure 5:**
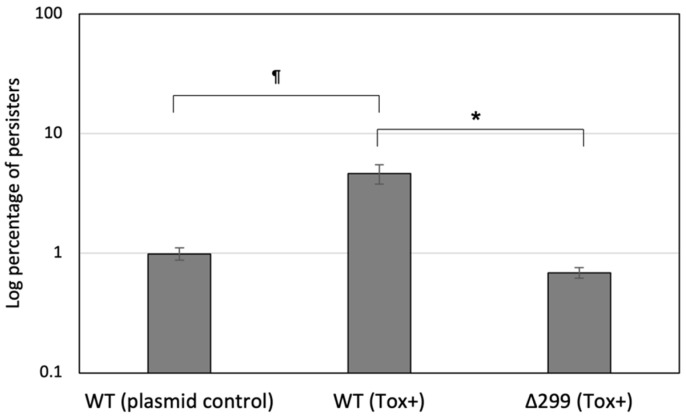
Effect of *tox40* overexpression on persister formation. Cultures of WT strain and Δ299 mutant overexpressing the *tox40* gene (Tox+) were treated with a lethal dose of ofloxacin to kill all nonpersisters. Cultures of WT harboring an empty plasmid were used as control. Aliquots of cells were withdrawn at the introduction of the antibiotic (T = 0) and after the 5-h antibiotic treatment. Cells were washed with PBS, serially diluted, and spot-plated onto THYE agar for CFU determination. The data (normalized to T = 0) are the average of three independent experiments ± standard error. ^¶^ denotes statistically significant difference compared to WT (plasmid control). * denotes statistically significant difference between Δ299 (Tox+) vs. WT (Tox+).

Work previously done by some members of our group showed that genes encoding toxin–antitoxin (TA) systems were important contributors to the formation of persisters in *S. mutans* [23,43,44]. We thus hypothesized that *pep299* could contribute to the formation of antibiotic persisters via the activation of specific TA system(s). Using the CSP-enriched persister population, we examined the toxin gene expression of MazEF, RelBE, and SmuATR systems as these TAs have been shown to significantly increase the levels of antibiotic persisters when overexpressed. We also measured the toxin expression level of two uncharacterized TA modules (SMU.40/41; SMU.218/219) identified using the TADB database [45]. The gene SMU.40 encoding a putative toxin of a novel type II TA system was the only toxin gene found specifically expressed (~4.5-fold) in persisters-CSP vs. normal-CSP. Interestingly, the gene SMU.40 (*tox40*) was also found to be highly activated (approximately eight-fold) following exposure to the DNA damage conditions known to activate *pep299* gene expression. In light of these findings, we next investigated the effect of *tox40* on persister formation using our classical persister assay. The toxin gene with its putative promoter region was cloned into pIB166 and the construct transferred into WT strain and its Δ299 mutant. Our results showed that whereas WT(Tox+) produced an increased (approximately five-fold) number of persisters, the inducible persistence phenotype was abolished for the Δ299(Tox+) mutant (Figure 5). Using qRT-PCR, we confirmed that *tox40* gene was significantly repressed (greater than three-fold) in Δ299 mutant vs. WT suggesting that *pep299* was required for the activation of the *tox40* gene in the CSP-induced persister population.

## 4. Discussion

Antibiotic persisters are considered a major cause of the recurrence of chronic bacterial infections. It is thus not surprising that over the last two decades many studies have focused on investigating the triggers and pathways of persister formation [42]. Many studies aiming at identifying persister genes were conducted using *E. coli* as a model. The screening of non-redundant knockout and expression libraries showed that several genes differing in function could influence persister formation [24]. Importantly, the screening of single gene knockout libraries did not result in the identification of mutants unable to produce persisters clearly indicating redundant pathways for their formation. Similar conclusions have been drawn from the genetic screening of expression libraries in *S. mutans* as there is more likely to be an apparent redundancy of persister genes [23].

Two types of antibiotic persisters have been described [26]. Spontaneous persistence occurs at very low rates and stochastic processes result in the generation of persisters. One such example relates to the type II TA systems [24], although the role of these systems in antibiotic persisters is still a debated question [42]. Typically, type II TAs are organized in an operon of two genes encoding a stable toxin that disrupts an essential cellular function and its labile antitoxin to counteract its toxic activity [46]. Several studies showed that the stochastic increase in the free toxin level could stimulate the formation of persisters [47]. We also showed that ectopic expression of toxin genes from MazEF, RelBE, and SmuATR type II TAs exerted an increase in persister level in *S. mutans* [23,44]. The formation of triggered persisters occurs following the exposure to stress or QS signaling molecules. Because stress-induced persisters are formed at a greater frequency upon the induction of bacterial stress response programs, a situation encountered during the treatment of infections, triggered persistence is thus generally more commonly studied. In this study, we were interested at investigating the role of genes encoding class II bacteriocins containing a highly conserved GG motif in the formation of triggered persisters. The recruitment of bacteriocins during genetic competence via QS is a recurring theme among streptococci [10], however, the involvement of bacteriocins in QS-induced persisters has not been thoroughly investigated. *S. mutans* is one of the most prolific producers of class II bacteriocins in the oral cavity [14]. These antimicrobial peptides function to kill competitors by dissipating the proton motive force via pore formation in the cytoplasmic membrane of target cell [16]. We identified an uncharacterized gene, *pep299*, that was specifically activated in the triggered persister population while being strongly repressed in proliferating normal cells. The gene *pep299* belongs to the core genome of *S. mutans* [48] and was predicted to encode a putative bacteriocin containing a canonical GG motif before the core peptide for its secretion into the extracellular environment. Our results did not demonstrate a role for Pep299 in the bacteriocin-mediated lysis of target cells using S-Pep299 and the two most commonly bacterial indicators for bacteriocins of Gram-positive bacteria [49]. It must be emphasized that a lack of detectable activity does not imply that *pep299* does not encode a functional bacteriocin. It is possible that Pep299 is a narrow spectrum bacteriocin and the susceptible bacterial targets have yet to be identified. Nevertheless, our results were not unexpected as several streptococcal peptide pheromones (e.g., competence-stimulating peptides) that share most features with bacteriocins have no or poor antimicrobial activity [50]. Instead of acting as a killing peptide, our results suggested that Pep299 may function as a secreted signaling peptide to mount a specific response by forming persisters to avoid eradication by the antibiotic treatment. The fact that *pep299* locus does not encode an immunity factor also reinforced this hypothesis.

In this study, we showed that the *pep299* gene was a main actor involved in the formation of triggered persisters via the intracellular QS system. More importantly, the ectopic gene expression of *pep299* was able to stimulate the formation of triggered persisters in the absence of the QS stimulus. This result would suggest that *pep299* might act as a downstream stimulator of the persistence phenotype. Interestingly, our results suggested that *pep299* exerts its effects via activation of a dedicated type II TA, the SMU.40/41 system. Although further studies would be required to confirm the mode of action of the toxin, bioinformatic analysis showed that Tox40 belongs to the ParE toxin family. These toxins affect DNA replication through arresting the enzyme DNA gyrase. In *E. coli*, the expression of ParE toxins induces the SOS response [51]. Since the SMU.40/41 TA was induced upon DNA damage, it can be speculated that the streptococcal toxin may have similar molecular mechanisms underlying its toxicity.

Our results strongly suggest that Pep299 was secreted into the extracellular environment since an overexpressing construct was able to induce the formation of persisters in cells unable to produce the peptide themselves. The Pep299 propeptide contains a GG motif for its export; we thus speculated that it could be exported by an ABC transporter such as the ComAB and/or NlmTE transporters [5]. In *S. mutans*, the ComAB and NlmTE systems are responsible for the secretion of CSP pheromone and nonlantibiotic bacteriocins, respectively. Interestingly, preliminary work done in our lab demonstrated that Pep299+ was still able to induce the formation of persisters while expressed in a ΔcomAB or ΔnlmTE mutant suggesting that Pep299 is exported via a dedicated as-yet undescribed secretion system.

Higher levels of persisters were obtained in Pep299+ biofilms suggesting that bacteria living in high cell density and proximity in biofilms could favorably affect the outcome of signaling. Our results suggest that Pep299 was partly released in the surroundings while some remained attached to the bacteria. By producing and releasing Pep299 into the environment, the cooperative population is exposed to exploitation by cheaters. The fact that Pep299 remains partly attached to the producing bacteria may help protect the population against cheaters.

Persisters play a considerable role in recalcitrant biofilm infections. One of the early responses of host innate immunity is reactive oxygen species production in reaction to bacterial invasion. Streptococci thus encounter reactive oxygen species in abundance within the host, culminating in DNA damage [17]. Our results suggest that *S. mutans* uses a DNA-damage inducible gene as a persister-promoting factor. This study introduced a new role for genes encoding GG-type leader bacteriocins, perhaps as a general survival strategy to protect the species while in a vulnerable physiological state. Since GG-motif peptides are widespread in bacteria [16,39], we can speculate that the coupling of bacteriocin production to persister formation may not be an adaptation unique to *S. mutans*; most likely, clades of streptococci using the CSP-ComDE QS system [52] may also be exploiting a bacteriocin-mediated persister strategy to initiate an adaptive response as a means to survive environmental stresses.

## 5. Conclusions

Bacteria can survive highly competitive microbiota by modulating the expression of genes such as bacteriocin genes. Persister formation is a core strategy used by bacteria, ensuring survival of the bacterial population. In this study, we were interested at the interplay between QS and persister formation as most bacteria live in complex polymicrobial communities and rely on QS to share information. We focused our research on genes encoding GG-motif-containing bacteriocins controlled by the canonical QS system of streptococci, as some of them also display signaling activity. We discovered that the *pep299* gene was specifically expressed in the non-multiplying persister state following stimulation by the QS pheromone. Interestingly, in vitro co-culture systems showed that a cell–cell contact was not necessary for exchange of information between Pep299+ and Pep299-deficient cells. Further experiments would need to be performed to elucidate the exact mechanism of action of *pep299*. Nonetheless, our results suggest two signaling pathways transmitting information from the stress-inducible QS pheromone and *pep299* to factors (e.g., Tox40) that promote persister formation. One strategy in combatting antibiotic persisters lies in preventing their formation by interfering with mechanisms known to promote their formation. Although we focused our study on the oral pathogen *S. mutans*, we can expect that our results would apply as well to Gram-positive species that use a peptide-based QS system to control bacteriocin production.

## Figures and Tables

**Table 1:** *S. mutans* strains used in this study.

Strain	Genotype	Resistance *^a^*	Description	Reference
UA159	wild-type			ATCC
Δ299	ΔSMU.299	Ery or Spc	In-frame insertion-deletion	This study
Pep299+	ΔSMU.299(pS921)	Ery Cml	Ectopic expression of *pep299*	This study
WT(plasmid control)	UA159(pIB166)	Cml	Empty plasmid	This study
WT(Tox+)	UA159(pS1109)	Cml	Ectopic expression of *tox40*	This study
Δ299(plasmid control)	ΔSMU.299(pIB166)	Spc Cml	Empty plasmid	This study
Δ299(Tox+)	ΔSMU.299(pS1109)	Spc Cml	Ectopic expression of *tox40*	This study

*^a^* Ery, Spc, Cml = resistance to erythromycin, spectinomycin and chloramphenicol, respectively.

## Data Availability

Not applicable.

## Supplementary Materials

The following supporting information can be downloaded at: https://www.mdpi.com/article/10.3390/genes13081434/s1, Table S1: Oligonucleotides used in this study.
