# Supplementary material for: A DNA-Damage Inducible Gene Promotes the Formation of Antibiotic Persisters in Response to the Quorum Sensing Signaling Peptide in Streptococcus mutans
Source: Genes (Basel). 2022 Aug 12;13(8):1434. doi: 10.3390/genes13081434 (PMC9408444; doi:10.3390/genes13081434)
Supplement: Supplementary file 1 [file genes-13-01434-s001.zip › genes-1843139-supplementary.pdf]

Table S1. Oligonucleotides used in this study.

	Forward primer	Reverse primer
**Insertion-deletion mutagenesis**		
Erythromycin resistance cassette	5′-**GGCGCGCC**CCGGGCCCAAAATTTGTTTGAT-3′	5′-**GGCCGGCC**AGTCGGCAGCGACTCATAGAAT-3′
Spectinomycin resistance cassette	5′-**GGCGCGCC**ACTAATAACGTAACGTGACTGGC-3′	5′-**GGCCGGCC**AAGTAGTTTCCGATATGGACGAG-3′
Gene SMU.299 (upstream)	5′-ATCTGAATGGAGCTAGAATG-3′	5′-**GGCGCGCC**CCCAGTTACATGTGAAAGTG-3′
Gene SMU.299 (downstream)	5′-**GGCCGGCC**GTAGTAAATAGCTGGGCTTC-3′	5′-AGGAAACCATGGAATCTGCT-3′
**Ectopic expression in pIB166**		
Pep299+	5′-**CCGCGG**GCAGATGTCATTAAGCGTAC-3′	5′-**GAATTC**GATGAGGTGTTTGCCGTTTA-3′
Tox+	5′-**CCGCGG**AAATAAAAAATGATTAATTGAGAAC-3′	5′-**GAATTC**CTATTAACATCATTTTACTAAAG-3′
**qRT-PCR**		
SMU.299	5′-ACGATGGAGCTAATGGCTAT-3′	5′-AAGCGTAAGCGGCAAAACTT-3′
SMU.40	5′-GACAATACAAGACAGCAAAGC-3′	5′- GCTACAATAATCAGCCCCTC-3′
SMU.150	5′-GATGTAATGGACAGCCAAACAC-3′	5′-TAACAAGAGTCGCACCTGCCAG-3′
SMU.151	5′-GGAGTAAACAAGCTGCTGATACG-3′	5′-TACAGATCCAACCGCACCAACTCC-3′
SMU.167	5′-ACGATGAACGTCAGCAGCAA-3′	5′-TTTTCCAAAACGTGGGTCAAC-3′
SMU.173	5′-GATCCCAAACAAGGACATGAAC-3′	5′-GGATAATCACGCTTGGTATTGC-3′
SMU.219	5′-AGGCAGACACTTAGTCACCT-3′	5′-GGCAAAAGTGTTGCTTTGGC-3′
SMU.423	5′-TTGAGGGTGGTGGTATGATTAG-3′	5′-CCAACGACTGGGAGAGTAACTG-3′
SMU.895	5′-GGTGGAGAGGCATTAGAGAACT-3′	5′-TCTTCTAAAATAATCCCATTGG-3′
SMU.1902	5′-CCTAGCCACTCTCTTGGATCA-3′	5′-CATGTAAAGTCATTGACAGAA-3′
SMU.1905	5′-AAAACTTCCCGCTGCAATTGTA-3′	5′-AAATATTAAATACTGATGCATTGG-3′
SMU.1914	5′-AAGAGCTCCTCCGATTCC-3′	5′-TAGGTGCTGGGCAAGGTTA-3′
gyrA (HKP)	5′-ATTGTTGCTCGGGCTCTTCCAG-3′	5′-ATGCGGCTTGTCAGGAGTAACC-3′

Restriction sites are in bold.
